# The Modulation of Laser Refractive Surgery on Sensory Eye Dominance of Anisometropia

**DOI:** 10.1155/2020/3873740

**Published:** 2020-03-27

**Authors:** Hongting Liu, Qi Chen, Fangfang Lan, Yan Luo, Enwei Lin, Wuqiang Luo, Ming Kong, Jiangxia Wang, Fengju Zhang

**Affiliations:** ^1^Beijing Tongren Eye Center, Beijing Tongren Hospital, Capital Medical University, Beijing Ophthalmology &Visual Sciences Key Lab, Beijing 100730, China; ^2^Guangxi Optometry and Visual Science Center, The People's Hospital of Guangxi Zhuang Autonomous Region, Nanning 530021, China; ^3^Biostatistics Department, School of Public Health, Johns Hopkins University, Baltimore, MD, USA

## Abstract

**Purpose:**

To evaluate the effect of laser refractive surgery on sensory eye dominance of anisometropia.

**Methods:**

A total of 156 subjects with nonanisometropic myopia and 70 subjects with anisometropic myopia were enrolled in the first part of the study. The dichoptic motion coherence threshold technique was applied to collect the normal dataset and distribution of sensory eye dominance. The second part of the study included 40 subjects with nonanisometropic myopia and 40 subjects with anisometropic myopia who received the femtosecond laser-assisted *in situ* keratomileusis (Fs-LASIK). A comprehensive ophthalmologic evaluation was performed with particular attention to sensory eye dominance preoperatively and one-week and one-month postoperatively. The ocular dominance index (ODI) was applied to evaluate the subject's overall degree of sensory ocular dominance. Visual acuity, sighting eye dominance, and stereo acuity were also accessed.

**Results:**

In experiment one, the mean ODI in the nonanisometropic group and the anisometropic group was 1.48 ± 0.63 and 1.95 ± 1.07, respectively. The ODI values of the anisometropic group were significantly higher than those of the nonanisometropic group (Mann–Whitney *U* test, *P* < 0.001). The demographics information and the distribution of ODI values in both groups are summarized in tables and figures. In experiment two, all LASIK procedures were uneventful and no postoperative complications were observed during the postoperative follow-up. Preoperatively, the ODI values of the anisometropic LASIK group were significantly higher than those of the nonanisometropic LASIK group, which was consistent with the results of part 1. However, one week after operation, the mean ODI values of the anisometropic LASIK group had significantly decreased from 1.89 ± 1.09 to 1.39 ± 0.44. And, the mean ODI values slightly increased to 1.65 ± 0.61 one-month postoperatively. In the nonanisometropic LASIK group, there were no statistically significant differences of ODI changes among preoperative, post-one-week and post-one-month visits. The demographics information and the changes of ODI of both LASIK groups are summarized in tables and figures.

**Conclusion:**

Stronger sensory eye dominance is seen in the subjects with anisometropic myopia compared to subjects with nonanisometropic myopia. The strong sensory dominance of anisometropia becomes more balanced at one week of postoperation but returns to the preoperative level after one month. Laser refractive surgery had a short-term modulation of sensory eye dominance.

## 1. Introduction

Laser refractive surgery is an effective method for patients with refractive errors to achieve spectacle/contact lens independence. The safety, efficacy, stability, and predictability of laser refractive surgeries have been widely studied in the past two decades [1]. It is very common for anisometropia [2] and also a useful treatment option for anisometropic amblyopia [3].

However, postoperative binocular vision disorders such as asthenopia, diplopia, and strabismus have been reported since the era of radial keratotomy (RK) [4–6].

Also, these disorders are more likely to occur if there was a preexisting binocular abnormality such as anisometropia or phoria/tropia before surgery [7].

It has been confirmed that binocular deficits of anisometropia include aniseikonia [8], poor stereovision [9], and imbalanced sensory ocular dominance (SED) [10]. Laser refractive surgeries eliminated aniseikonia and improved the stereo acuity of anisometropia [11], but a paucity of data exists regarding the changes of sensory dominance after the operations in the literature.

Different from the sighting dominance that was clinically accessed by the hole-in-card test, the neural basis of SED is that one eye usually has a larger weighted contribution than the contralateral eye when the information of two eyes is combined in the visual cortex [12]. It is mainly a reflection of intraocular suppression at cortical perception level, a strong SED means a strong intraocular suppression and accounts for the poor visual acuity and stereovision in anisometropia with or without amblyopia [13, 14]. The extent of sensory eye dominance can be quantitatively measured with the aid of the laboratory-based psychophysics technique [15–17].

It would be interesting to evaluate the changes of sensory eye dominance of anisometropia after LASIK surgeries, as it will provide a further understanding of postoperative binocular vision-related complaints. In the light of sensory eye dominance which has been served as the model of neural plasticity and can be modulated through refractive correction [17], occlusion [18], or perception learning [19], our study may also provide a new treatment approach for binocular visual disorders after laser surgeries.

## 2. Participants and Methods

### 2.1. Participants

As the SED measurement has not been translated to wider clinical practice, in the first part of the experiment, we collected the normative data of sensory eye dominance using the dichoptic motion coherence threshold measurement in a clinical setting. A total of 156 subjects with nonanisometropic myopia and 70 subjects with anisometropic myopia were enrolled. The inclusion criteria for myopia subjects were best-corrected visual acuity (VA) greater than or equal to 20/20 in each eye, refractive errors within ±8.00 diopter (D) sphere and ±2.00 D cylinder, and no history of ocular diseases. Anisometropia was defined as an interocular refractive error difference (IRED) equal to or greater than 1.00 D.

In the second part of the experiment, 40 subjects with nonanisometropic myopia and 40 subjects with anisometropic myopia that received femtosecond laser-assisted *in situ* keratomileusis (Fs-LASIK) were enrolled initially, followed by a one-week and a one-month postoperative visit. The inclusion criteria were patients who were voluntary candidates for refractive surgery; aged 18 to 40 years; and had refractive errors within ±8.00 diopter (D) sphere and ±2.00 D cylinder; no history of ocular diseases; absence of any systemic disorder; and absence of any obvious ocular deviation.

Patients must have anisometropia of 1.00 D or more to be included in the anisometropic group. Patients with known contraindication for corneal refractive surgery and patients with a residual refractive error of more than 0.75 D or vision acuity lower than 20/25 at one month after surgery were excluded from the study.

The study followed the tenets of the Declaration of Helsinki and was approved by the Institutional Review Board/Ethics Committee of the People's Hospital of Guangxi Zhuang Autonomous Region. Informed consent was obtained from all subjects.

### 2.2. Methods

For the first part of the experiment, the routine ophthalmology examinations were performed with particular attention to sensory eye dominance. For the second part of the experiment, the thorough ophthalmologic evaluations were conducted including laser refractive surgery eligibility, sighting dominance, sensory dominance, and stereoacuity.

#### 2.2.1. Sighting Dominance Measurement

The hole-in-card test was used to determine sighting dominance. The participants were instructed to keep both eyes open while holding a card with both hands and viewing a 6-m distant target through a hole in the middle of the card. They were asked to alternately close each eye to determine the dominant viewing eye. Observers were then instructed to slowly draw the card back toward their head without changing the previously aligned position. The eye that was underneath the hole in the card was considered to be the dominant eye.

#### 2.2.2. Sensory Dominance Measurements

**Figure 1:**
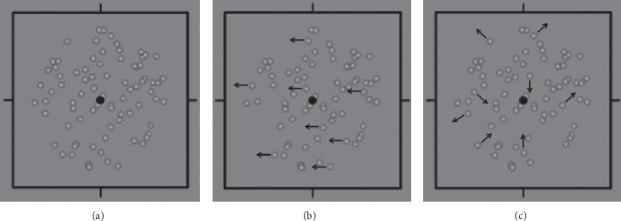
Motion coherence threshold measurement. (a) shows a representative image of the sensory dominance test, and (b) and (c) are schematic images, with the dots moving to the left constitute the signal dot population in the right eye, while the dots moving in random directions constitute the noise population. The arrows in (b) and (c) are for illustration purposes and were not presented in the tests. (a) Binocular perception. (b) Right eye. (c) Left eye.

Sensory dominance was measured by the dichoptic motion coherence threshold test. The detailed stimuli and task setting parameters have been previously described [13, 20–22]. In brief, the stimuli were dichoptically presented by a pair of polarizing glass, and the test was conducted on a desktop computer using Matlab and PsychToolBox. In each trial, one eye was presented with a population of signal dots that all moved in the same direction (left, right, up, or down); the fellow eye was presented with the noise dot that moved in random directions. The task was to indicate the motion direction of the signaled dots (Figure 1). Task difficulty was controlled by varying the relative proportion of signal to noise in the display. The signal-to-noise ratio at which task performance reached approximately 80%, as determined by a 3-down and 1-up staircase strategy, was known as the motion coherence threshold for each eye. Both eyes were tested separately, and the eye with lower motion detection threshold has higher sensory dominance.

To quantitatively evaluate a subject's overall degree of ocular dominance, the ocular dominance index (ODI) is applied. It is calculated as the ratio of motion coherence threshold from two eyes. A ratio (ODI value) of 1 indicates a complete sensory balance between the eyes. The farther the ODI is from 1, the stronger the sensory dominance. The experiment was programmed with the commercial software (Matlab, Version 2012Rb; the Math Works, Natick, MA, and Psychophysics Toolbox, Version 3) provided by Guangdong Nuoyide Biomedical Technology Development Co., Ltd.

#### 2.2.3. Stereovision Measurement

**Figure 2:**
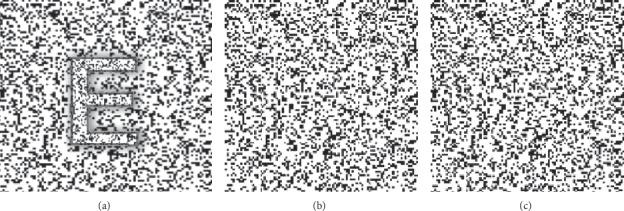
A random-dot pattern for stereo discrimination (depth perception). (a) shows a representative image of a stereovision test. (b) and (c) are the images presented separately for each eye. (a) Binocular perception. (b) Right eye. (c) Left eye.

Stereovision was accessed with computerized random-dot stereograms provided by Guangdong Nuoyide Biomedical Technology Development Co., Ltd. A pair of polarizing glass was applied to allow different stimuli to be presented to each eye. The task was to indicate the direction of the “E “(Figure 2). Test results were graded from one to four grades, with the corresponding stereovision as 400” (arcsec), 300”, 200”, and 100”, respectively.

#### 2.2.4. Laser *In Situ* Keratomileusis Operations

LASIK procedure was performed with the STAR S4 IR excimer laser system (Abbott Medical Optics, Inc., California, USA). The flaps were created using the IntraLase femtosecond laser system (Abbott Medical Optics, Inc., California, USA) with a superior hinge of 110-*μ*m thicknesses. Emmetropia was selected as the target refraction to minimize preoperative errors.

#### 2.2.5. Statistical Analysis

Analyses were performed using SPSS statistical package (Version 22.0; IBM SPSS Inc., Chicago, Illinois) and STATA (Version 16.0; StataCorp LP, College Station, TX).

Mann–Whitney *U* test was applied to compare the data between groups due to the non-normal distribution of the data. Kappa test was applied to evaluate the consistency of sighting and sensory eye dominance measurements. A linear mixed-effect model was applied to evaluate the sensory eye dominance changes after the operations, with the ODI values as the dependent variable and the interaction term between groups and visits as the independent variable. A random intercept was included to account for the correlation among the repeated measures from the same subject. A *P* value of less than 0.05 was considered statistically significant.

## 3. Results

**Figure 3:**
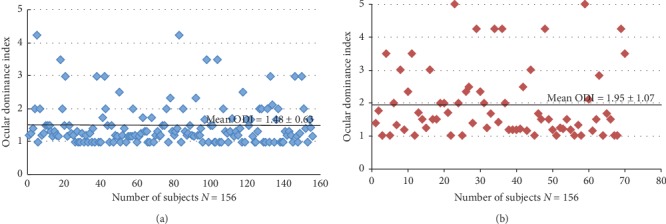
The ODI distribution in two groups. (a) ODI distribution of the nonanisometropia group. (b) ODI distribution of the anisometropia group.

1. A total of 156 subjects in the nonanisometropia group and 70 subjects in the anisometropia group were recruited in the first part of the study. The demographic information, the intraocular refraction differences, and the ODI differences of the two groups are summarized in Table 1. There were no significant differences between the two groups in terms of age, gender, mean spherical equivalent, and mean cylinder equivalent. The mean ODI in the nonanisometropia group and the anisometropia group was 1.48 ± 0.63 and 1.95 ± 1.07, respectively. The ODI values were significantly higher in the anisometropia group than in the nonanisometropia group (Mann–Whitney *U* test, *P* < 0.001), i.e., subjects with anisometropia have stronger sensory ocular dominance in comparison to subjects with nonanisometropia. The distribution of ODI differences between the nonanisometropia group and the anisometropia group is shown in Figure 3.
2. The relationship of sensory dominant eye and sighting dominant eye was also explored in both the groups and is shown in Table 2. The sensory dominant eye was defined as the eye with a lower threshold in the dichoptic motion coherence threshold test. Forty-one subjects in the nonanisometropia group and 12 subjects in the anisometropia group were excluded because of the thresholds in both eyes (ODI = 1), i.e., these subjects had complete balanced sensory perception. Thus, 115 subjects from the nonanisometropia group and 58 subjects from the anisometropia group were included for analysis. A low but statistically significant correlation between sighting dominance and sensory dominance was found in the anisometropia group (kappa = 0.34, *P* < 0.05). However, there was no significant correlation in the nonanisometropia group (*P*=0.183).
3. In the second part of the experiment, 40 subjects with nonanisometropic myopia and 40 subjects with anisometropic myopia that received the FS-LASIK were enrolled initially. All surgical procedures were uneventful. Postoperatively, 34 subjects from the myopia group and 33 subjects from the anisometropia group completed the post-one-week visits. Six subjects from the nonanisometropia group and 7 subjects from the anisometropia group were excluded because of missing the visits, residual refractive error more than 0.75D, or vision acuity lower than 20/25. At one month after the operation, 29 subjects from the myopia group and 31 subjects from the anisometropia group completed the visit. Five subjects from the nonanisometropia group and 2 subjects from the anisometropia group were excluded because of missing the visits or vision acuity lower than 20/25. The demographic information and the ODI changes of the two groups are shown in Table 3.

Preoperatively, the ODI of the anisometropia group was significantly higher than in the nonanisometropia group (*P*=0.022), which is consistent with the results of the first part of the study. At one week after the operations, the mean ODI of the anisometropia LASIK group significantly decreased from 1.89 to 1.39 and had no difference with the nonanisometropia LASIK group. At one month of postoperation, the mean ODI of the anisometropia LASIK group slightly increased from 1.39 to 1.65, but the differences were not statistically significant either compared with its previous visit or the nonanisometropia LASIK group, i.e., the sensory eye dominance of the anisometropia group became more balanced after LASIK surgeries.

**Figure 4:**
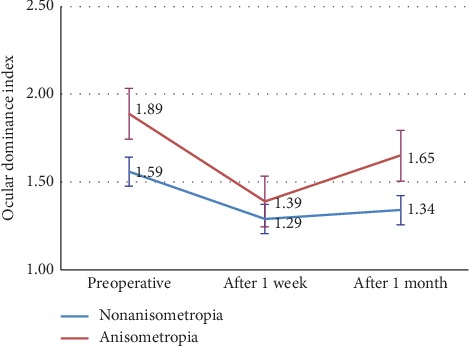
The ODI changes of two groups after LASIK operations.

In the nonanisometropia LASIK group, there were no statistical differences of ODI changes among preoperative and post-one-week and one-month visits. The changes of ODI in two groups are shown in Figure 4.

## 4. Discussion

The basic principle of measuring sensory eye dominance is to measure the perception threshold of each eye under dichoptic view and evaluate the dominance extent by the threshold ratio of the two eyes. However, the outputs of various laboratory methods are not usually the same as different visual stimuli such as grating, letters, noise patterns, or gabor spots, and different tasks such as phase integration, global direction discrimination, motion coherence discrimination, and letter discrimination were applied [10, 12, 16, 20].

In our study, the clinically available sensory dominance measurement is based on dichoptic motion coherence threshold technology. This technology demonstrated good test-retest reliability and had a high consistency with the modified Bagolini striated lens test. It was first reported by Li et al. [20] and applied in several other studies [13, 18, 23].

In the first part of the study, we collected the normative values and distributions of subjects with myopia. The mean and median ODI in the nonanisometropia group were 1.48 ± 0.63 and 1.25, respectively. It is comparable to the study results of Li et al. [20]; in which, the threshold ratio was between 1 and 1.6 for the majority of their subjects who have unclear dominance (61%) and over 1.8 for the rest of participants who have clear dominance. However, the sample size was relatively small (44 subjects) and subjects with anisometropia were not included.

In our study, there was a significantly higher ODI in the anisometropia group, with mean and median ODI 1.95 ± 1.07 and 1.5, respectively. It indicates that subjects with anisometropia have stronger ocular dominance in comparison to subjects with nonanisometropia. The results were consistent with the research findings of Jiang et al. [10].

In their study, the continuous flashing technique was applied to measure the ocular dominance. *t*-test was used to compare the intraocular difference, and *t*-value was used as the ODI. Hence, the median value of ODI was 4.59 for anisometropic myopia, which was significantly higher than that (3.12) of nonanisometropic myopia.

Although it was a different measurement and a different calculation method, their data showed that anisometropia subjects have stronger sensory dominance. They further examined the strength of ocular dominance and the amplitude of anisometropia by applying a cutting point of ODI. A subject with ODI < 2 was regarded as an unclear dominance and ODI ≤ 2 regarded as clear sensory dominance. In the subjects with clear dominance, a mild but significant correlation was further revealed between the strength of ocular dominance and the amplitude of anisometropia (*R* = 0.42 in myopic anisometropia and 0.62 in hyperopic anisometropia).

However, we did not further divide the clear or unclear dominance in the above way. The first part of the study aimed to collect the preliminary data of the measurements and to evaluate if it can distinguish the anisometropia subjects from the anisometropia subjects in a clinical setting. Once it was proved to be effectively detecting the sensory dominance differences between the two groups, we were able to continue, in the second part of the study, to observe the changes of sensory dominance of anisometropia and take the nonanisometropia group as a control after LASIK operations.

We further explored the relationship between sighting dominance and sensory dominance, as sighting dominance was recorded as part of the routine presurgery examinations. It was applied widely for a range of clinical decisions, such as monovision treatment [24], cataract surgeries [25], and contact lens wear.

A statistically significant but low correlation was found in the anisometropia group, but not in the nonanisometropia group. The lack or weak correlation between the two types of eye dominance was also seen in other studies [10, 20]. It was not surprising as they have different generating mechanisms and measuring methods. The sighting dominance is usually related to handedness and footedness, while sensory dominance is a reflection intraocular suppression of the cortex perception level; it is associated with binocularity.

In the second part of the study, the sensory dominance of the anisometropia group was much more balanced at one week of postoperation, with the mean ODI largely decreased from 1.89 to 1.39. It was a significant change compared to its preoperative level. Also, there were no differences when compared with the nonanisometropia group. However, the strong sensory dominance of anisometropia bounced back at one month of postoperation. The mean ODI of the anisometropia group increased from 1.39 to 1.65, with no differences compared to the preoperative level. In other words, the sensory dominance of the anisometropia group became more balanced after LASIK operations, but the balancing effects of operations gradually faded away within a month.

Sensory dominance has been served as the model for neural plasticity, even in adults. It has been proved that it can be modulated through patching [26], dichoptically viewing different video [27], refraction correction [17], or specific perception learning paradigm [19].

In the study that examined the effect of refraction correction on sensory dominance, compared with uncorrected anisometropia, the dominance imbalance was less severe in corrected anisometropia (at least 16 weeks of spectacle wearing). As it was a cross-sectional study, it estimated that an optical correction introduces the neuronal change at somewhere between 1 hour and 16 weeks of correction [17].

Similar to refraction correction, laser surgery is a method of rapid but permanent refraction correction. The abrupt refraction correction of laser surgeries eliminated the intraocular refraction difference of anisometropia and released the intraocular suppression, and the sensory dominance changes of LASIK operations can be detected within one month. Laser surgery introduced a midterm shift of sensory eye dominance in the anisometropia group.

In the study that the binocular phase combination paradigm to access the effect of LASIK surgery on the sensory dominance [28], it was implied that a long-term adaption period (16 weeks or more) is necessary to enable the surgery to be truly effective. However, 15 subjects were included in the study with the postoperative visits scattered from 8 days to 96 days. The changing trend of sensory dominance within one month maybe oversighted because of the small sample size and a broad visit window.

The clinical implications of our study would be in two aspects. First, it is well known that modulating intraocular suppression of sensory dominance can improve both binocular vision and monocular vision acuity in anisometropic amblyopia [21, 29, 30]. In the conditions that laser refractive surgeries were applied to treat the anisometropia amblyopia, we would like to propose the amblyopia training should start as early as one-week postoperatively or no later than a month. As indicated in our study, the sensory ocular dominance of anisometropia was most balanced during this time frame.

Secondly, there were individual subjects from both groups whose sensory dominance became ever stronger after the operation although there were no complaints of visual disturbance in our study. Considering the modulation effect of LASIK on sensory dominance, the scattered case reports of asthenopia, diplopia, and strabismus in the literature may associate with the adaption failure to the changes of sensory dominance at the cortical perception level. We may need to follow closely with the patients about the sensory eye dominance status, and if possible, specific perception training regimes such as push-pull training [31] can be applied as a potential treatment.

It has been reported that the stereovision of anisometropia improved after laser refractive surgeries [16]. We explored to see if it could be contributed to a more balanced sensory dominance. However, the changes of sensory eye dominance were not statistically related to the stereopsis in our study. Preoperatively, the stereovision was grade 4 (100”) for all except three subjects of the anisometric LASIK group. The stereovision of these 3 subjects improved from grade 3 (200”) and grade 2 (300”) to all grade 4 (100”) at post-one-week visit, with more balanced sensory dominance. The low screening rate was probably due to the rough grading of our stereovision test. The finest stereovision of our test was 100”, which merely equals to a moderate stereovision in the TNO test and the Butterfly stereo acuity test. Thus, the subtle changes of stereovision were not fully revealed.

The main limitation of the study was the short follow-up time. It would be interesting to follow-up until 3 months postoperatively to see if the balanced sensory dominance could retain at the month-one level or completely return back to the preoperative levels. But, the numbers of subjects at the one-month end reduced down to 29-30 in each group; the sample maybe not enough for 3 months of follow-up. Hence, further studies of a greater number of subjects with a longer follow-up are required to confirm these preliminary findings.

## Figures and Tables

**Table 1:** Demographic information and ODI values of subjects in part one.

Items	Nonanisometropia group	Anisometropia group
No. of subjects	156	70
Age (y)	25.97 ± 6.04	27.03 ± 7.20
Female (%)	85 (54.48)	40 (57.14)
Mean spherical equivalent (D)	−4.63 ± 1.82	−3.93 ± 2.10
Mean cylinder equivalent (D)	−0.70 ± 0.58	−0.70 ± 0.57
Intraocular refraction difference (D)	0.35 ± 0.32	2.05 ± 0.79
Mean ODI	1.48 ± 0.63	1.95 ± 1.07
Median ODI	1.25	1.5
Mann–Whitney *U* test	*Z* = −3.302, *P* < 0.001	

**Table 2:** The relationship of sensory dominance eye and sighting dominance eye.

	Nonanisometropia group (*n* = 115)		Anisometropia group (*n* = 58)	
	OD as sighting dominant eye	OS as sighting dominant eye	OD as sighting dominant eye	OS as sighting dominant eye
OD as sensory dominant eye	40	20	23	12
OS as sensory dominant eye	30	25	7	16
Kappa test	Kappa = 0.122, *P*=0.183		Kappa = 0.34, *P*=0.009	

**Table 3:** The demographics information and the changes of ODI after LASIK surgeries.

	Nonanisometropia group	Anisometropia group
Preoperative information		
Number of subjects	40	40
Age (y)	25.55 ± 6.83	26.41 ± 5.50
Female (%)	25 (62.5)	27 (67.5)
Mean spherical equivalent (D)	−4.91 ± 1.80	−4.04 ± 1.92
Mean cylinder equivalent (D)	−0.60 ± 0.50	−0.71 ± 0.55
Intraocular refraction difference (D)	0.41 ± 0.35	1.86 ± 0.64
Mean ODI	1.53 ± 0.66	1.89 ± 1.09
Sig. of ODI difference Comparison between groups	*Z* = 2.29, *P*=0.022^*∗*^	
One-week postoperative visit		
Number of subjects	34	33
Mean ODI	1.29 ± 0.33	1.39 ± 0.44
Sig. of ODI difference Compared to preoperative	*Z* = −1.60, *P*=0.11	*Z* = −4.04, *P* ≤ 0.001^*∗*^
Sig. of ODI difference Comparison between groups	*Z* = 0.11, *P*=0.91	
One-month postoperative visit		
Number of subjects	29	31
Mean ODI	1.34 ± 0.53	1.65 ± 0.61
Sig. of ODI difference Compared to preoperative	*Z* = −1.47, *P*=0.142	*Z* = −1.82, *P*=0.069
Sig. of ODI difference Comparison between groups	*Z* = 1.75, *P*=0.08	

## Data Availability

The datasets analyzed during the current study are available from the corresponding author on reasonable request.
